# Pre-operative Waterlow score and outcomes after kidney transplantation

**DOI:** 10.1186/s12882-022-02902-8

**Published:** 2022-08-04

**Authors:** Anna Brotherton, Felicity Evison, Suzy Gallier, Adnan Sharif

**Affiliations:** 1 Department of Nephrology and Transplantation, University Hospitals Birmingham, Queen Elizabeth Hospital, Edgbaston, Birmingham, B15 2WB UK; 2 Research Informatics, Research Development and Innovation, University Hospitals Birmingham, Queen Elizabeth Hospital, Birmingham, UK; 3 PIONEER - HDR-UK Health Data Hub in Acute Care, University of Birmingham, Birmingham, UK; 4 Institute of Immunology and Immunotherapy, University of Birmingham, Birmingham, UK

**Keywords:** Waterlow, Surrogate, Kidney transplantation, Length of stay, Readmission, Mortality

## Abstract

**Background:**

Waterlow scoring was introduced in the 1980s as a nursing tool to risk stratify for development of decubitus ulcers (pressure sores) and is commonly used in UK hospitals. Recent interest has focussed on its value as a pre-op surrogate marker for adverse surgical outcomes, but utility after kidney transplantation has never been explored.

**Methods:**

In this single-centre observational study, data was extracted from hospital informatics systems for all kidney allograft recipients transplanted between 1^st^ January 2007 and 30^th^ June 2020. Waterlow scores were categorised as per national standards; 0–9 (low risk), 10–14 (at risk), 15–19 (high risk) and ≥ 20 (very high risk). Multiple imputation was used to replace missing data with substituted values. Primary outcomes of interest were post-operative length of stay, emergency re-admission within 90-days and mortality analysed by linear, logistic or Cox regression models respectively.

**Results:**

Data was available for 2,041 kidney transplant patients, with baseline demographics significantly different across Waterlow categories. As a continuous variable, the median Waterlow score across the study cohort was 10 (interquartile range 8–13). As a categorical variable, Waterlow scores pre-operatively were classified as low risk (*n* = 557), at risk (*n* = 543), high risk (*n* = 120), very high risk (*n* = 27) and a large proportion of missing data (*n* = 794). Median length of stay in days varied significantly with pre-op Waterlow category scores, progressively getting longer with increasing severity of Waterlow category. However, no difference was observed in risk for emergency readmission within 90-days of surgery with severity of Waterlow category. Patients with ‘very high risk’ Waterlow scores had increased risk for mortality at 41.9% versus high risk (23.7%), at risk (17.4%) and low risk (13.4%). In adjusted analyses, ‘very high risk’ Waterlow group (as a categorical variable) or Waterlow score (as a continuous variable) had an independent association with increase length of stay after transplant surgery only. No association was observed between any Waterlow risk group/score with emergency 90-day readmission rates or post-transplant mortality after adjustment.

**Conclusions:**

Pre-operative Waterlow scoring is a poor surrogate marker to identify kidney transplant patients at risk of emergency readmission or death and should not be utilised outside its intended use.

**Supplementary Information:**

The online version contains supplementary material available at 10.1186/s12882-022-02902-8.

## Introduction

**Fig. 1:**
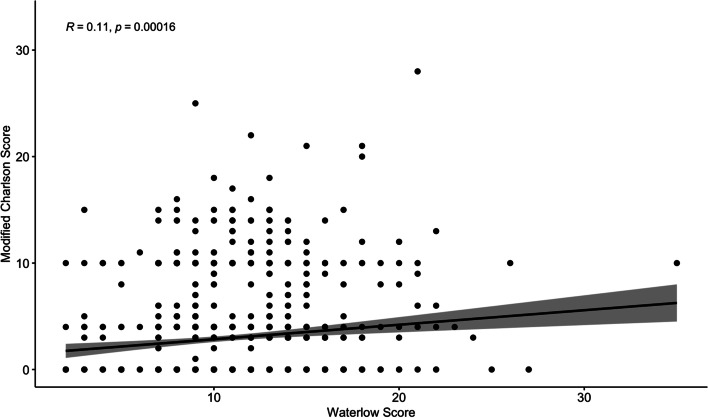
Correlation matrix between Waterlow score and modified Charlson score

Pre-operative risk prediction tools undertaken prior to major surgery can support counselling and consent processes and aid the development of targeted intra- and post-operative care. Several scoring systems have been established and validated in the context of general surgery with variable sensitivity [1]. One example is the Waterlow score, first introduced in the 1980s as a nursing tool to stratify patients at risk for development of decubitus ulcers (pressure sores) [2]. Using a multisystem approach, with weighted scores based on several variables (Fig. 1), patients are categorised into ‘at risk’, ‘high risk’ or ‘very high risk’ for developing decubitus ulcers. Due to its ease of use, it is commonly utilised by UK hospital staff for this specific purpose and forms part of a standard nursing assessment for acute medical and surgical admissions [3–5]. However, there is conflicting data with regards to the association between Waterlow scores and development of pressure ulcers, with positive [6] versus negative [7–9] reports of its predictive ability. Some of the criticism labelled at Waterlow scoring is the inter-rater variability, poor concordance and low sensitivity that limits generalizability.

While the original creation of the Waterlow score was never intended be used as a predictive tool [10], due to its ubiquitous assessment by surgical nurses it has been frequently assessed as a surrogate measure to predict adverse clinical outcomes after surgery. In a systematic review of the general surgery literature, summary evidence suggests high pre-operative Waterlow scores (> 20) correlate with increased risk for morbidity, mortality, length of stay and intensive care unit (ICU) admission [11]. Increasing Waterlow scores are also strongly associated with post-operative infection risk after neck of femur fractures [12]. From a transplant perspective, Khambalia and colleagues have shown high pre-operative Waterlow scores are associated with total length of hospital stay and intensive care unit length of stay following simultaneous pancreas and kidney (SPK) transplantation [13]. No study has explored Waterlow scores in the context of kidney-alone transplantation, with the only solid-organ transplant outcome data in the literature limited to length of stay in the work from Khambalia et al. To date, no universally accepted pre-operative risk prediction tool exists in the context of solid organ transplantation.

Considering its ubiquitous use in pre-operative settings, it is likely Waterlow scores may be perceived as an acceptable surrogate measure to predict adverse outcomes after surgery. In the setting of transplantation, no data exist to show any association between Waterlow scores and adverse clinical outcomes independent of other important factors. Therefore, the aim of this study was to study the association between Waterlow scores on admission for kidney transplant candidates and post-operative outcomes including length of stay, risk of emergency re-hospitalisation within 90-days of surgery and mortality.

## Materials and methods

### Study population

We undertook a retrospective cohort analysis of all consecutive adult kidney-alone transplants performed at a single-center between 1^st^ January 2007 and 30^th^ June 2020. Our only exclusion criterion was transplantation of multiple organs; all other kidney allograft recipients were eligible for inclusion. Data were electronically extracted by the Department of Health Informatics for every study recruit, with manual data linkage to additional electronic patient records. This study is reported according to the RECORD (REporting of studies Conducted using Observational Routinely-collected health Data) statement (12) and the STROBE reporting guidelines (13).

### Determination of Waterlow scores and categories

The Waterlow score is derived after assessment and point allocation across several items: build/weight, height, visual assessment of the skin, sex/age, continence, mobility, and appetite, and special risk factors, divided into tissue malnutrition, neurological deficit, major surgery/trauma, and medication. Potential scores range from 1 to 64 on a continuous scale, with Waterlow scores categorised as follows; 0–9 (low risk), 10–14 (at risk), 15–19 (high risk) and ≥ 20 (very high risk). For this analysis, both continuous and categorical variables were used for assessing the association of Waterlow scores and adverse outcomes.

### Definition of variables

We utilised existing pre-determined ethnicity classifications, as obtained from electronic patient records. Ethnicity was classified into the following categories: White, Black, South Asian (also referred to as Indo-Asian) and “other”; patients where this was not recorded were excluded from the analysis of ethnicity. Determination of socioeconomic deprivation was based upon the Index of Multiple Deprivation (IMD), a multiple deprivation model calculated at the local level area, as utilized by the UK Government. The IMD is a composite construct of seven domains reflective of area’s socioeconomic deprivation, namely: 1) Income Deprivation, 2) Employment Deprivation, 3) Health Deprivation and Disability, 4) Education Skills and Training Deprivation, 5) Barriers to Housing and Services, 6) Living Environment Deprivation, and 7) Crime. The resulting IMDs are then divided into national quintiles, with quintile one being the most deprived, and quintile five the least deprived. Charlson co-morbidity index was calculated with removal of diabetes mellitus and chronic kidney disease constituents.

### Statistical analysis

For this analysis, we analysed Waterlow scores both as a continuous and categorical variable. The primary outcomes of interest were post-operative length of stay (in days), emergency re-admission within 90-days and mortality in relation to Waterlow scores.

Categorical data was presented as numbers and percentages, with continuous variables reported as medians and interquartile ranges (IQRs). Differences between groups were compared using chi-square tests or Fisher’s Exact tests for categorical variables and student t-tests or Mann–Whitney tests (parametric or non-parametric data respectively) for all continuous variables. Correlation between Waterlow and Charlson scores, both considered surrogate measures of cumulative morbidity, was checked, and reported as the correlation coefficient (*R*value). Missing data was handled by multiple imputation using predictive mean matching [14]. The number of multiple imputations was planned to be greater than the degree of missing data [15]. The imputation model utilised all variables used in the analysis model (including the outcome variable) to ensure congeniality between the imputation and analysis models. A sensitivity analysis was done without propensity score analysis and missing Waterlow score data simply excluded.

Frailty variable was treated as both ordinal – low risk, at risk, high risk and very high risk – and continuous variables in the analysis models. Length of stay and emergency readmission within 90-days of surgery was assessed by linear and logistic regression respectively (reported as Odds Ratio [OR] with 95% Confidence Intervals [CI]). Assumptions checked for linear regression modelling include confirmation of a linear relationship, minimal autocorrelation, and homoscedasticity. Unadjusted survival analyses were performed by generation of Kaplan–Meier curve estimates. Adjusted time-to-survival outcomes were analysed with the Cox’s Proportional Hazards Model. The proportional hazard assumption was checked and satisfied by examination of plots of the log-negative-log of the within-group survivorship functions versus log time as well as comparing Kaplan–Meier (observed) with Cox (expected) survival curves with our study variables, alongside selected covariables for adjusted analyses (reported as Hazard Ratios [HR] with 95% CI). All regression models were adjusted for age, sex, ethnicity, waiting time, donor type, socio-economic deprivation status, Charlson comorbidity score and diabetes, with the inclusion of Waterlow scores as an ordinal (model 1) and continuous (model 2) variable.

Statistical analysis was performed using R version 4.0.4 (R Foundation for Statistical Computing, Vienna, Austria). A *p* value < 0.05 was deemed to be of statistical significance.

### Approvals

This study received institutional approval and was registered as an audit (audit identifier; CARMS-12578). The study was conducted in accordance with the Declaration of Helsinki ethical standards. Formal participant consent was not required for this study as it utilized anonymized pre-existing data from electronic health records. The corresponding author had full access to all data.

## Results

### Study cohort

Data was available for 2,041 kidney transplant patients, with baseline demographics shown in Table 1 and showing significant differences in baseline demographics across Waterlow categories. As a continuous variable, the median Waterlow score across the study cohort was 10 (interquartile range 8–13). As a categorical variable, Waterlow scores pre-operatively were classified as low risk (*n* = 557), at risk (*n* = 543), high risk (*n* = 120), very high risk (*n* = 27) and a large proportion of missing data (*n* = 794). Therefore, a multiple imputation model was created as described in the methods section and generated the following categories: low risk (*n* = 931), at risk (*n* = 881), high risk (*n* = 186), very high risk (*n* = 43). This model was utilised for subsequent analyses.

**Table 1:** Baseline study demographics

Variable	Low Risk *N* = 557	At Risk *N* = 543	High Risk *N* = 120	Very High Risk *N* = 27	Missing data *N* = 794	*P* value
Age in years (median ± interquartile range)	44.0 ± 13.0	50.3 ± 13.9	53.7 ± 12.2	55.7 ± 12.2	45.7 ± 13.8	< 0.001
Male sex (%)	377 (67.7%)	266 (49.0%)	73 (60.8%)	17 (63.0%)	474 (59.7%)	< 0.001
White ethnicity (%)	324 (58.2%)	346 (63.7%)	61 (50.8%)	18 (66.7%)	568 (71.5%)	< 0.001
Diabetes (%)	15 (2.8%)	74 (14.2%)	46 (40.4%)	9 (34.6%)	87 (11.0%)	< 0.001
Body Mass Index (kg/m^2^)	26.8 ± 8.0	28.0 ± 5.2	28.7 ± 5.2	28.9 ± 5.6	27.8 ± 6.99	0.007
Time waitlisted (days)	1109 ± 1036	1179 ± 994	1217 ± 999	1535 ± 1436	950 ± 872	< 0.001
Charlson Score	2.59 ± 4.23	2.99 ± 4.59	3.52 ± 4.82	5.78 ± 6.32	3.79 ± 5.48	< 0.001

### Association between Waterlow score and Charlson co-morbidity score

The correlation between Waterlow and Charlson scores is shown in Fig. 1, highlighting a statistically significant but weak correlation between the two measures (*R* = 0.11, *p* < 0.001).

### Length of stay, emergency hospitalization and mortality (unadjusted)

**Fig. 2:**
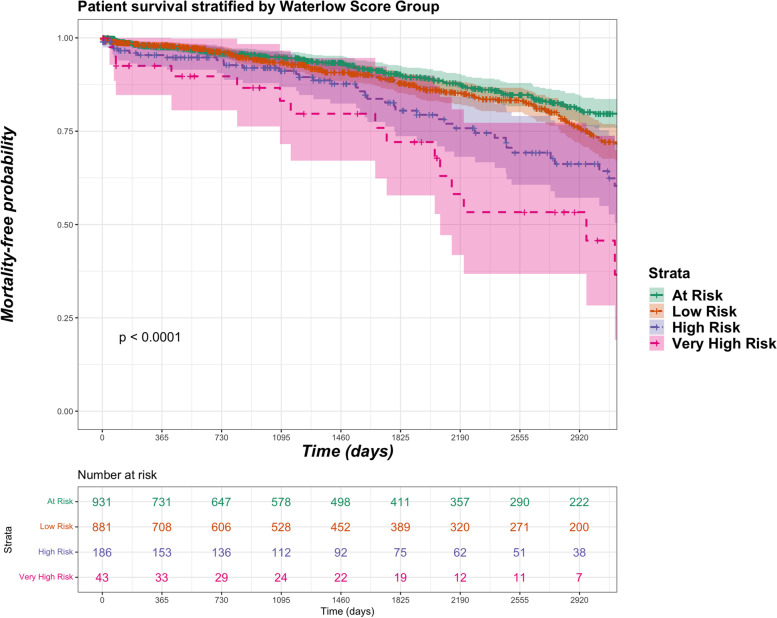
Kaplan-Meir survival for study cohort stratified by pre-op Waterlow category (multiple imputation model)

Median length of stay in days (± interquartile range) varied significantly with pre-op Waterlow category scores, progressively getting longer with increasing severity of Waterlow category; low risk (8 ± 5), at risk (9 ± 6), high risk (10 ± 7) and very high risk (11 ± 7) (*p* = 0.0242). However, no difference was observed in risk for emergency readmission within 90-days of surgery with severity of Waterlow category; low risk (36.3%), at risk (39.2%), high risk (36.6%) and very high risk (45.0%) (*p* = 0.5731). Patients with ‘very high risk’ Waterlow scores had increased risk for mortality at 41.9% versus high risk (23.7%), at risk (17.4%) and low risk (13.4%). Figure 2 shows the unadjusted Kaplan-Meir plot for mortality stratified by Waterlow category for the study cohort.

### Adjusted survival analyses

The association between Waterlow group (categorical variable) and Waterlow score (continuous variable) with post-operative length of stay in days are shown in Table 2. On univariable analysis, ‘high risk’ and ‘very high risk’ Waterlow risk categories and Waterlow score as a continuous variable were associated with increased length of stay after transplant surgery. On multivariable analysis, only ‘very high risk’ Waterlow risk category and Waterlow score as a continuous variable remained associated with increased length of stay after transplant surgery.

**Table 2:** Waterlow group (as categorical variable) or Waterlow score (as continuous variable) and association with post-op length of stay in days after kidney transplantation

Variable		Odds Ratio (95% CI)	*P* value	Odds Ratio (95% CI)	*P* value
		**Univariable analysis**		**Multivariable analysis**^a^	
Waterlow group	Low Risk	REF	-	REF	-
	At Risk	1.361 (0.614–3.017)	0.448	1.116 (0.477–2.610)	0.800
	High Risk	5.352 (1.373–20.864)	0.016	2.577 (0.585–11.135)	0.211
	Very High Risk	67.916 (4.834–954.108)	0.002	17.892 (1.117–286.641)	0.042
Waterlow score		1.216 (1.092–1.354)	< 0.001	1.137 (1.005–1.286)	0.041

^a^Regression model includes waterlow group/score, age, sex, ethnicity, waiting time, Charlson score, diabetes status, donor type, socio-economic status

The association between Waterlow group (categorical variable) and Waterlow score (continuous variable) with emergency 90-day emergency readmission rates are shown in Table 3. In both univariable and multivariable analyses, no significant association was observed with any Waterlow risk category or Waterlow score as a continuous variable with emergency 90-day readmission rates.

**Table 3:** Waterlow group (as categorical variable) or Waterlow score (as continuous variable) and association with emergency 90-day readmission after kidney transplantation

Variable		Odds Ratio (95% CI)	*P* value	Odds Ratio (95% CI)	*P* value
		**Univariable analysis**		**Multivariable analysis**^a^	
Waterlow group	Low Risk	REF	-	REF	-
	At Risk	1.028 (0.848–1.246)	0.777	0.909 (0.725–1.139)	0.408
	High Risk	1.011 (0.725–1.401)	0.947	0.940 (0.633–1.385)	0.757
	Very High Risk	1.599 (0.858–2.955)	0.134	1.374 (0.674–2.781)	0.377
Waterlow score		1.018 (0.992–1.045)	0.165	1.013 (0.981–1.047)	0.418

^a^Regression model includes waterlow group/score, age, sex, ethnicity, waiting time, Charlson score, diabetes status, donor type, socio-economic status

Finally, the association between Waterlow group (categorical variable) and Waterlow score (continuous variable) with mortality are shown in Table 4. While in univariable analysis all Waterlow risk categories and Waterlow score as a continuous variable were associated with increased risk for mortality, they were both no longer significant in multivariable analysis.

**Table 4:** Waterlow group (as categorical variable) or Waterlow score (as continuous variable) and association with mortality after kidney transplantation

Variable		Hazard Ratio (95% CI)	*P* value	Hazard Ratio (95% CI)	*P* value
		**Univariable analysis**		**Multivariable analysis**^a^	
Waterlow group	Low Risk	REF	-	REF	-
	At Risk	1.394 (1.099–1.768)	0.006	1.002 (0.760–1.322)	0.988
	High Risk	2.013 (1.426–2.842)	< 0.001	1.253 (0.833–1.885)	0.278
	Very High Risk	4.130 (2.515–6.784)	< 0.001	1.606 (0.906–2.846)	0.105
Waterlow score		1.095 (1.066–1.125)	< 0.001	1.033 (0.999–1.069)	0.057

^a^Regression model includes waterlow group/score, age, sex, ethnicity, waiting time, Charlson score, diabetes status, donor type, socio-economic status

Full tables of regression analysis are shown in Supplementary Tables 1, 2 and 3. In a non-propensity score matched cohort sensitivity analysis with missing Waterlow score data simply excluded, no difference was observed in the outcomes of the regression analyses (data not shown).

## Discussion

In this large single-centre analysis, we observed many kidney failure patients being admitted for kidney transplant surgery have raised Waterlow risk categories when checked pre-operatively as part of routine nurse admission checks. As a surrogate measure for risk stratification, ‘very high risk’ Waterlow group (as a categorical variable) or Waterlow score (as a continuous variable) had an independent association with increase length of stay after transplant surgery. No association was observed between any Waterlow risk group or score with emergency 90-day readmission rates. When analysed alone any Waterlow risk group or Waterlow score was associated with increased risk for post-transplant mortality. However, after adjustment with baseline variables, no association remained with either Waterlow risk groups or Waterlow score and mortality. Our analysis raises some interesting discussion points about the utility and value of using Waterlow risk score, or other surrogate measures of ‘fitness’, suggesting caution in their utilisation as a proxy measure for adverse outcomes that is outside of their intended application.

As a recommended tool for pressure ulcer risk stratification among acute medical and surgical admissions [16], the simplicity of Waterlow scores has made them commonplace in the United Kingdom. However, compliance rates for adherence to Waterlow score charting are variable across reports [11]. One of the major limitations in our study was the significant number of missing data with regards to Waterlow scores pre-operatively, which may be related to inadequate completion pre-operatively or an informatics data capture issue. These are unlikely to be missing at random as illustrated in Table 1, with missing participants tending to be at the low risk end of the spectrum for most baseline demographics. Inclusion after multiple imputation may be skewing the median Waterlow scores artificially higher. Indeed, the analysis performed without including missing data shows even less importance of the Waterlow score and may be more accurate.

The other major issue with the Waterlow score is studies tend to report variable inter-rater reliability due to the presence of subjective variables [17]. This is a concern as it questions the generalisability of the Waterlow score between different assessments and/or assessors.

The greatest issue identified in this analysis is the questionable utility of Waterlow scores as a surrogate measure for identifying kidney transplant candidates at higher risk for adverse post-transplant outcomes. While intuitively the subjective components of Waterlow scoring can render higher grades based upon personal bias, the failure of Waterlow scores to identify adverse post-transplant outcomes independent of other variables (apart from prolonged hospital stays post-operatively for ‘very high risk’ patients) demonstrates its inadequacy as a risk predictor. However, that was never its intended use. Even in its original context of risk stratification of patients at risk for pressure sores, the superiority of Waterlow scores versus rival scoring systems is not clear. In a prospective single centre study, Gurkan et al. compared the predictive capacity of three pressure sore risk tools in 250 patients undergoing abdominal surgery: Norton, Braden and Waterlow risk assessment scales [18]. They observed the Waterlow score demonstrated the best values of predictive validity among the three scales in the assessment of pressure injury risk, with sensitivity (100%), specificity (48.1%), positive predictive value (20.8%) and negative predictive value (100%) demonstrated for the Waterlow score (a cut-off point of 10). However, all three scales had low specificity despite high sensitivity in terms of a good risk prediction. In a narrative review of 26 studies, Charamboulous et al. confirmed the predictive validity of the Waterlow is characterised by high specificity and low sensitivity, with inadequate inter-rater reliability due to lack of clear definitions within categories and differentiating level of user knowledge [17].

Our results are important as published literature extrapolating use of Waterlow score from risk assessment of pressure sores to adverse post-surgical outcomes has seemingly favourable evidence. In a systematic review of published evidence in the surgical literature, Nayar and colleagues identified 4 relevant studies (*n*= 505) exploring morbidity and mortality associated with Waterlow score in surgical patients [11]. The studies included general, vascular, transplant and orthopaedic surgical settings. A high Waterlow score demonstrated a statistically significant association with increased morbidity, length of stay, need for intensive care admission and mortality. Furthermore, this was a more accurate predictor for adverse post surgery outcomes compared to others scoring systems in routine practice (Portsmouth Physiological and Operative Severity Score for the enumeration of Mortality [P-POSSUM] or American Society of Anesthesiologists [ASA]. However, these handful of studies have several limitations including small cohort sizes, heterogenous cut-offs for dichotomous Waterlow scores and lack of adjustment against baseline characteristics suggesting publication bias that should be interpreted with caution.

Rather than relying on non-validated tools, better measures for risk stratification are warranted to identify kidney transplant candidates at high risk for adverse outcomes. One possibility is assessment for frailty, a syndrome of accelerated ageing across multiple physiological systems and characterised by increased vulnerability to stressors, which has been studied in the context of surgical interventions including kidney transplantation. While associated with several adverse outcomes after kidney transplantation [19], several limitations exist in its clinical application. Firstly, frailty can be defined using many tools and it is unclear which is best from a plethora of available options [20]. This is important as frailty scoring tools vary in their requirement of time, resource, and equipment. Secondly, frailty is a dynamically changing status and evolves with time for patients living with kidney failure [21]. Therefore, a quick and simple tool that can be utilised prior to time-pressured kidney transplant surgery will have most utility and clinical application. Further work in this area is important to understand how assessment of frailty can be used for pre-operative risk stratification.

A more fundamental question is what benefit such risk stratification has for kidney transplant candidates. Unless changes to surgical intervention or post-operative care will occur, which currently lacks any evidence-base, apart from risk counselling it serves no additional benefit for the success of that kidney transplant in the peri- and post-operative period. By contrast, it may persuade some transplant professionals to deny the opportunity for kidney transplantation to some kidney failure patients identified as high-risk who will still have survival benefits from proceeding with kidney transplantation versus remaining on dialysis. In the absence of any tailored intervention to attenuate risk, the aim and purpose of any risk stratification before kidney transplant surgery requires careful thought for utility. While we currently lack robust evidence-based intervention to demonstrate ‘prehabilitation’ of frail kidney transplant candidates has clinical benefits, encouraging work is happening in this area. McAdmas-DeMarco et al. conducted a single-arm intervention trial (with a historical control as comparison) in 18 kidney transplant candidates using centre-based prehabilitation involving weekly physical therapy sessions [22]. Physical activity based on accelerometry improved by 64% (*p* = 0.004) and length of hospital admission was shorter for 5 participants who received transplantation compared to historic demographic-matched controls (5 versus 10 days respectively; *RR* = 0.69; 95% CI 0.50–0.94; *p* = 0.02). However, while high satisfaction was reported by the 18 participants, it is important to note 84/106 eligible kidney transplant candidates decline to participate (with 6/24 consented participants not actually participating). Therefore, feasibility on a wide scale will require more engagement from patients to be worthwhile. Our analysis also suggests if such a strategy is employed, rather than using surrogate measures like Waterlow score intended for other purposes, we must adopt validated risk stratifications measures that are amendable to intervention with a stratified management plan.

Limitations of our work must be appreciated for an accurate interpretation of the data. The study was retrospective in nature, and so is prone to all the shortcomings of such analyses, including the inability to establish causation, and the potential effects of unmeasured or intangible confounders. As previously highlighted, we had a sizable proportion of missing data with regards to pre-operative Waterlow scores which is a major limitation. While we undertook a multiple imputation model to overcome this, the limitations of this statistical approach must be acknowledged [14]. In addition, we have highlighted the literature regarding inter-rater variability for pre-operative Waterlow scores. This is important to appreciate in our dataset as it spans 13.5 years and scores are likely to have been conducted by a heterogenous group of nurses.

To conclude, in this large single-centre study, we observed over half of kidney transplant candidates admitted for transplant surgery with available data had elevated Waterlow risk scores. As a nursing tool designed to risk stratify acutely admitted patients for pressure sores, as a surrogate measure for physiological fitness in isolation it is associated with prolonged length of stay after transplantation and mortality (but not emergency 90-day readmissions post-operatively). However, after adjustment against baseline demographics, most of these associations are lost. Our data suggest Waterlow scores have no utility as a risk stratification tool for adverse post-transplant outcomes outside its intended use. Further research is required to validate risk stratification measures to identify high-risk kidney transplant candidates, although the purpose for adopting such pathways should be to modify peri- and post-operative care to improve outcomes rather than deny the opportunity for kidney transplantation to kidney failure candidates.

## Supplementary Information

**Additional file 1.** STROBE Statement—Checklist of items that should be included in reports of ***cohort studies***.

**Additional file 2:** **Supplementary Table 1.** Complete linear regression analysis of Waterlow grading and length of stay (in days) after kidney transplantation. **Supplementary Table 2.** Complete logistic regression analysis of Waterlow grading and emergency 90-day readmission after kidney transplantation. **Supplementary Table 3.** Complete Cox proportional hazard regression analysis of Waterlow grading and mortality after kidney transplantation.

## Data Availability

The data that support the findings of this study are not publicly available due to legal restrictions but available from the corresponding author on reasonable request.

## References

[1] National Institute for Health and Care Excellence. Perioperative care in adults. Evidence review for preoperative risk stratification tools. Accessed at; https://www.niceorguk/guidance/ng180/evidence/c-preoperative-risk-stratification-tools-pdf-8833151056. 32931173

[2] Waterlow J (1991). A policy that protects. The Waterlow Pressure Sore Prevention/Treatment Policy. Prof Nurse.

[3] Thompson D (2005). An evaluation of the Waterlow pressure ulcer risk-assessment tool. Br J Nurs.

[4] Thorn CC, Smith M, Aziz O, Holme TC (2013). The Waterlow score for risk assessment in surgical patients. Ann R Coll Surg Engl.

[5] Wang JW, Smith P, Sarker SJ (2019). Can Waterlow score predict 30-day mortality and length of stay in acutely admitted medical patients (aged >/=65 years)? Evidence from a single centre prospective cohort study. BMJ Open.

[6] Serpa LF, de Gouveia Santos VL, Gomboski G, Rosado SM (2009). Predictive validity of Waterlow Scale for pressure ulcer development risk in hospitalized patients. J Wound Ostomy Continence Nurs.

[7] Kottner J, Dassen T, Tannen A (2009). Inter- and intrarater reliability of the Waterlow pressure sore risk scale: a systematic review. Int J Nurs Stud.

[8] Kottner J, Dassen T (2010). Pressure ulcer risk assessment in critical care: interrater reliability and validity studies of the Braden and Waterlow scales and subjective ratings in two intensive care units. Int J Nurs Stud.

[9] Walsh B, Dempsey L (2011). Investigating the reliability and validity of the waterlow risk assessment scale: a literature review. Clin Nurs Res.

[10] Waterlow JA (2005). Waterlow assessment: not a predictor. Br J Nurs.

[11] Nayar SK, Li D, Ijaiya B, Lloyd D, Bharathan R (2021). Waterlow score for risk assessment in surgical patients: a systematic review. Ann R Coll Surg Engl.

[12] El-Daly I, Ibraheim H, Culpan P, Bates P (2015). Pre-operative Waterlow score: Predicts risk of post-operative infection in patients with neck of femur fractures. Injury.

[13] Khambalia HA, Moinuddin Z, Summers AM (2015). A prospective cohort study of risk prediction in simultaneous pancreas and kidney transplantation. Ann R Coll Surg Engl.

[14] Sterne JA, White IR, Carlin JB (2009). Multiple imputation for missing data in epidemiological and clinical research: potential and pitfalls. BMJ.

[15] Hayati Rezvan P, Lee KJ, Simpson JA (2015). The rise of multiple imputation: a review of the reporting and implementation of the method in medical research. BMC Med Res Methodol.

[16] Pressure ulcers: prevention and management. Clinical guideline [CG179]. Published: 23 April 2014. Accessed at; https://www.niceorguk/guidance/cg179/chapter/1-recommendations.

[17] Charalambous C, Koulori A, Vasilopoulos A, Roupa Z (2018). Evaluation of the Validity and Reliability of the Waterlow Pressure Ulcer Risk Assessment Scale. Med Arch.

[18] Gurkan A, Kirtil I, Aydin YD, Kutuk G (2022). Pressure injuries in surgical patients: a comparison of Norton, Braden and Waterlow risk assessment scales. J Wound Care.

[19] Quint EE, Zogaj D, Banning LBD (2021). Frailty and Kidney Transplantation: A Systematic Review and Meta-analysis. Transplant Direct.

[20] Dent E, Kowal P, Hoogendijk EO (2016). Frailty measurement in research and clinical practice: A review. Eur J Intern Med.

[21] Chu NM, Deng A, Ying H (2019). Dynamic Frailty Before Kidney Transplantation: Time of Measurement Matters. Transplantation.

[22] McAdams-DeMarco MA, Ying H, Van Pilsum RS (2019). Prehabilitation prior to kidney transplantation: Results from a pilot study. Clin Transplant.

