# Supplementary material for: Pre-operative Waterlow score and outcomes after kidney transplantation
Source: BMC Nephrol. 2022 Aug 4;23:273. doi: 10.1186/s12882-022-02902-8 (PMC9351155; doi:10.1186/s12882-022-02902-8)
Supplement: Supplementary file 2 — Additional file 2: Supplementary Table 1. Complete linear regression analysis of Waterlow grading and length of stay (in days) after kidney transplantation. Supplementary Table 2. Complete logistic regression analysis of Waterlow grading and emergency 90-day readmission after kidney transplantation. Supplementary Table 3. Complete Cox proportional hazard regression analysis of Waterlow grading and mortality after kidney transplantation. [file 12882_2022_2902_MOESM2_ESM.docx]

**Supplementary Table 1. Complete linear regression analysis of Waterlow grading and length of stay (in days) after kidney transplantation**

| **Variable** | | **Waterlow group** | | **Waterlow score** | |
| --- | --- | --- | --- | --- | --- |
|  |  | **Odds Ratio (95% CI)** | **P value** | **Odds Ratio (95% CI)** | **P value** |
| Waterlow group | Low Risk | REF | - |  | |
|  | At Risk | 1.116 (0.477-2.610) | 0.800 |  |  |
|  | High Risk | 2.577 (0.585-11.135) | 0.211 |  |  |
|  | Very High Risk | 17.892 (1.117-286.641) | 0.042 |  |  |
| Waterlow score | |  | | 1.137 (1.005-1.286) | 0.041 |
| Age | | 1.027 (0.997-1.058) | 0.074 | 1.025 (9.995-1.056) | 0.104 |
| Sex | Female | REF | - | REF | - |
|  | Male | 1.647 (0.751-3.611) | 0.213 | 1.766 (0.809-3.856) | 0.154 |
| Ethnicity | White | REF | - | REF | - |
|  | Asian | 2.016 (0.712-5.710) | 0.187 | 2.104 (0.745-5.944) | 0.161 |
|  | Black | 6.504 (1.321-32.017) | 0.021 | 6.280 (1.278-30.878) | 0.024 |
|  | Other | 0.592 (0.121-2.903) | 0.518 | 0.583 (0.119-2.861) | 0.507 |
|  | Unknown | 7.536 (0.231-246.129) | 0.256 | 7.137 (0.219-232.446) | 0.269 |
| Waiting time (days) | | 1.001 (1.001-1.001) | <0.001 | 1.001 (1.001-1.001) | <0.001 |
| Charlson Score | | 1.562 (0.973-1.147) | 0.192 | 1.059 (0.975-1.149) | 0.174 |
| Recipient diabetes | | 1.046 (0.281-3.889) | 0.946 | 0.956 (0.255-3.586) | 0.947 |
| Donor type | Donation after brain death | REF | - | REF | - |
|  | Donation after cardiac death | 3.380 (1.087-10.511) | 0.036 | 3.401 (1.095-10.568) | 0.035 |
|  | Living | 0.321 (0.131-0.788) | 0.013 | 0.326 (0.133-0.799) | 0.014 |
| Index of multiple deprivation | 1 (Most deprived) | REF | - | REF | - |
|  | 2 | 0.811 (0.276-2.377) | 0.702 | 0.817 (0.279-2.395) | 0.713 |
|  | 3 | 0.571 (0.180-1.807) | 0.340 | 0.580 (0.183-1.836) | 0.354 |
|  | 4 | 2.165 (0.601-7.792) | 0.238 | 2.122 (0.590-7.631) | 0.250 |
|  | 5 (Least deprived) | 0.268 (0.070-1.022) | 0.054 | 0.268 (0.070-1.021) | 0.054 |

**Supplementary Table 2. Complete logistic regression analysis of Waterlow grading and emergency 90-day readmission after kidney transplantation**

| **Variable** | | **Waterlow group** | | **Waterlow score** | |
| --- | --- | --- | --- | --- | --- |
|  |  | **Odds Ratio (95% CI)** | **P value** | **Odds Ratio (95% CI)** | **P value** |
| Waterlow group | Low Risk | REF | - |  | |
|  | At Risk | 0.909 (0.725-1.139) | 0.408 |  |  |
|  | High Risk | 0.940 (0.633-1.385) | 0.757 |  |  |
|  | Very High Risk | 1.374 (0.674-2.781) | 0.377 |  |  |
| Waterlow score | |  | | 1.013 (0.981-1.047) | 0.418 |
| Age | | 1.010 (1.002-1.018) | 0.017 | 1.008 (1.001-1.017) | 0.032 |
| Sex | Female | REF | - | REF | - |
|  | Male | 0.922 (0.750-1.135) | 0.443 | 0.942 (0.768-1.159) | 0.576 |
| Ethnicity | White | REF | - | REF | - |
|  | Asian | 1.116 (0.851-1.462) | 0.425 | 1.127 (0.860-1.475) | 0.384 |
|  | Black | 1.459 (0.972-2.183) | 0.067 | 1.447 (0.965-2.164) | 0.072 |
|  | Other | 0.602 (0.371-0.946) | 0.033 | 0.603 (0.372-0.947) | 0.033 |
|  | Unknown | 0.299 (0.069-0.905) | 0.057 | 0.298 (0.069-0.903) | 0.056 |
| Waiting time (days) | | 1.000 (<1.000-1.000) | 0.169 | 1.000 (<1.000-1.000) | 0.181 |
| Charlson Score | | 1.017 (0.995-1.039) | 0.124 | 1.017 (0.996-1.039) | 0.117 |
| Recipient diabetes | | 0.742 (0.520-1.051) | 0.097 | 0.705 (0.492-1.000) | 0.052 |
| Donor type | Donation after brain death | REF | - | REF | - |
|  | Donation after cardiac death | 0.780 (0.580-1.046) | 0.099 | 0.781 (0.580-1.047) | 0.100 |
|  | Living | 0.634 (0.499-0.804) | <0.001 | 0.640 (0.504-0.811) | <0.001 |
| Index of multiple deprivation | 1 (Most deprived) | REF | - | REF | - |
|  | 2 | 0.855 (0.642-1.135) | 0.280 | 0.852 (0.640-1.130) | 0.268 |
|  | 3 | 0.869 (0.640-1.178) | 0.368 | 0.870 (0.640-1.179) | 0.371 |
|  | 4 | 0.958 (0.682-1.340) | 0.803 | 0.953 (0.679-1.333) | 0.779 |
|  | 5 (Least deprived) | 0.720 (0.496-1.035) | 0.079 | 0.718 (0.495-1.033) | 0.077 |

**Supplementary Table 3. Complete Cox proportional hazard regression analysis of Waterlow grading and mortality after kidney transplantation**

| **Variable** | | **Waterlow group** | | **Waterlow score** | |
| --- | --- | --- | --- | --- | --- |
|  |  | **Hazard Ratio (95% CI)** | **P value** | **Hazard Ratio (95% CI)** | **P value** |
| Waterlow group | Low Risk | REF | - |  | |
|  | At Risk | 1.002 (0.760-1.322) | 0.988 |  |  |
|  | High Risk | 1.253 (0.833-1.885) | 0.278 |  |  |
|  | Very High Risk | 1.606 (0.906-2.846) | 0.105 |  |  |
| Waterlow score | |  | | 1.033 (0.999-1.069) | 0.057 |
| Age | | 1.057 (1.046-1.069) | <0.001 | 1.057 (1.045-1.069) | <0.001 |
| Sex | Female | REF | - | REF | - |
|  | Male | 1.092 (0.854-1.397) |  | 1.103 (0.864-1.408) | 0.433 |
| Ethnicity | White | REF | - | REF | - |
|  | Asian | 0.754 (0.536-1.059) | 0.103 | 0.765 (0.545-1.073) | 0.121 |
|  | Black | 0.784 (0.467-1.316) | 0.357 | 0.780 (0.465-1.307) | 0.344 |
|  | Other | 0.665 (0.308-1.437) | 0.299 | 0.679 (0.315-1.462) | 0.322 |
|  | Unknown | 1.877 (0.578-6.097) | 0.295 | 1.840 (0.568-5.957) | 0.309 |
| Waiting time (days) | | 1.000 (<1.000-1.000) | 0.691 | 1.000 (<1.000-1.000) | 0.665 |
| Charlson Score | | 1.014 0.991-1.037) | 0.239 | 1.014 (0.992-1.037) | 0.217 |
| Recipient diabetes | | 1.760 (1.278-2.425) | <0.001 | 1.705 (1.246-2.355) | 0.001 |
| Donor type | Donation after brain death | REF | - | REF | - |
|  | Donation after cardiac death | 0.855 (0.596-1.225) | 0.392 | 0.858 (0.599-1.228) | 0.402 |
|  | Living | 0.668 (0.499-0.893) | 0.006 | 0.677 (0.507-0.905) | 0.008 |
| Index of multiple deprivation | 1 (Most deprived) | REF | - | REF | - |
|  | 2 | 0.782 (0.555-1.102) | 0.160 | 0.778 (0.552-1.097) | 0.152 |
|  | 3 | 0.967 (0.676-1.381) | 0.852 | 0.969 (0.678-1.385) | 0.864 |
|  | 4 | 0.840 (0.576-1.227) | 0.367 | 0.827 (0.567-1.205) | 0.322 |
|  | 5 (Least deprived) | 0.697 (0.439-1.105) | 0.124 | 0.704 (0.444-1.116) | 0.135 |
